# Synergy of Pd Clusters and TiO_2_ {101} for Photocatalytic Nonoxidative Coupling of Methane

**DOI:** 10.1002/advs.202524322

**Published:** 2026-07-13

**Authors:** Jianlong Yang, Annika S. Tang, Junwang Tang

**Affiliations:** ^1^ Industrial Catalysis Center Department of Chemical Engineering Tsinghua University Beijing China; ^2^ Yulin Innovation Institute of Clean Energy Yulin China; ^3^ Department of Engineering Science University of Oxford Oxford UK

**Keywords:** {101} facet‐exposed TiO_2_, C_2_ hydrocarbons, methane, nonoxidative coupling, Pd single atoms and clusters

## Abstract

Photocatalytic nonoxidative coupling of methane (NOCM) offers a sustainable route to valorize CH_4_ into C_2_ hydrocarbons, yet it is impeded by low activity, poor stability, and overoxidation. We demonstrate a {101} facet‐exposed TiO_2_ photocatalyst decorated with coexisting Pd single atoms and clusters, which achieves a high C_2_H_6_ production rate of 600 µmol·h^−1^·g^−1^ at room temperature with near 100% selectivity and stoichiometric H_2_ co‐production. The catalyst exhibits exceptional durability, maintaining stable performance over 30 h in a flow reactor. This enhanced performance stems from efficient interfacial charge transfer between the {101} facet and the coexisting Pd single atoms and clusters. Moreover, stable three‐fold coordinated surface oxygen atoms suppress lattice oxygen involvement, preventing overoxidation and ensuring high selectivity. This work overcomes key thermodynamic and kinetic limitations, presenting a robust design strategy for efficient photocatalytic methane conversion.

## Graphical abstract

This study presents photocatalytic nonoxidative coupling of methane over cal‐Pd_0.05_/TiO_2_{101}, achieving near 100% selectivity and long‐term stability under mild flow conditions through synergistic single atoms and clusters catalysis.

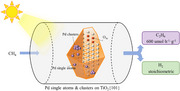

## 1 Introduction

The shale gas revolution has rendered methane (CH_4_) an abundant, low‐cost feedstock [1]. However, commercial valorization relies on energy‐intensive, multi‐step processes, mainly high temperature steam reforming (∼700 °C) followed by Fischer–Tropsch synthesis (230–450 °C, 2–5 MPa) [2, 3, 4]. These routes suffer from high capital costs, CO_2_ emissions, and coking issues [5]. Consequently, the development of efficient, economical methods for the direct conversion of methane under mild conditions remains an urgent and significant challenge.

Photocatalytic methane coupling presents a promising alternative route for upgrading CH_4_, encompassing both the oxidative (OCM) and nonoxidative coupling pathways (NOCM). OCM is thermodynamically favorable due to the presence of oxygen and typically has a higher C_2_ yield [6]. For instance, a recent Au/TiO_2_ photocatalyst achieved a CH_4_ conversion rate of 1.1 mmol h^−1^ with ∼90% C_2_ selectivity [7]. However, overoxidation to CO_2_ remains a major drawback, driven by the persistent presence of superoxide radicals, reducing atomic utilization efficiency.

In contrast, photocatalytic NOCM employs CH_4_ as the sole reactant, achieves 100% atomic utilization efficiency with H_2_ as the only byproduct. This inherently leads to high product selectivity. However, the overall yield currently remains low due to the thermodynamic unfavourability of the reaction (ΔG = +68.6 kJ/mol). The effective activation of stable C─H bond requires photocatalysts with deep valence bands, typically metal oxides featuring O 2p orbitals (e.g., TiO_2_ [8], BiVO_4_ [9], ZnO [10]). Unfortunately, this often results in the involvement of lattice oxygen (O_L_) during the reaction, which can over‐oxidize the desired products and degrade the catalyst structure. For example, Pd_1_/TiO_2_ has shown a notable C_2_H_6_ yield of 2.76 µmol·h^−1^ [11], but suffered significant performance decay within 6 h. These limitations highlight the critical challenge of simultaneously enhancing both activity and durability in photocatalytic CH_4_ conversion systems.

Therefore, precise control over surface structure, such as crystal facet engineering and interface construction, is crucial [6]. Among various photocatalysts, TiO_2_ stands out as an ideal platform due to its tunable nanostructure and amenability to surface modification [12]. Early studies revealed that three‐fold‐coordinated bridging O (O_3c_) atoms on the {101} surface are more stable than two‐fold‐coordinated bridging O (O_2c_) atoms on the {001} surface [13]. The presence of stable O_L_ atoms is essential in NOCM in order to prevent its involvement in the over‐oxidation of products [11]. Moreover, charge transfer dynamics in TiO_2_ are strongly facet dependent: {001} facets preferentially facilitate hole transfer, while {101} facets favour electron transfer [14]. This anisotropic charge separation can be further enhanced by selectively depositing co‐catalysts on specific facets [15].

In this work, Pd single atoms and clusters were deposited on TiO_2_ with exposed {101} facets for photocatalytic NOCM in a flow reactor. Under light irradiation, the optimized cal‐Pd_0.05_/TiO_2_{101} catalyst shows a high C_2_H_6_ yield of 600 µmol·h^−1^·g^−1^, 33 times higher than that of TiO_2_ with exposed high‐energy {001} facets, with nearly 100% selectivity and stoichiometric H_2_ evolution. It has been found that Pd clusters function as a hole acceptor, synergizing with {101} facets to enhance charge separation. Meanwhile, the presence of stable three‐fold coordinated O atoms on the {101} surface contributes to high selectivity and long‐term stability.

## 2 Results and Discussion

**FIGURE 1:**
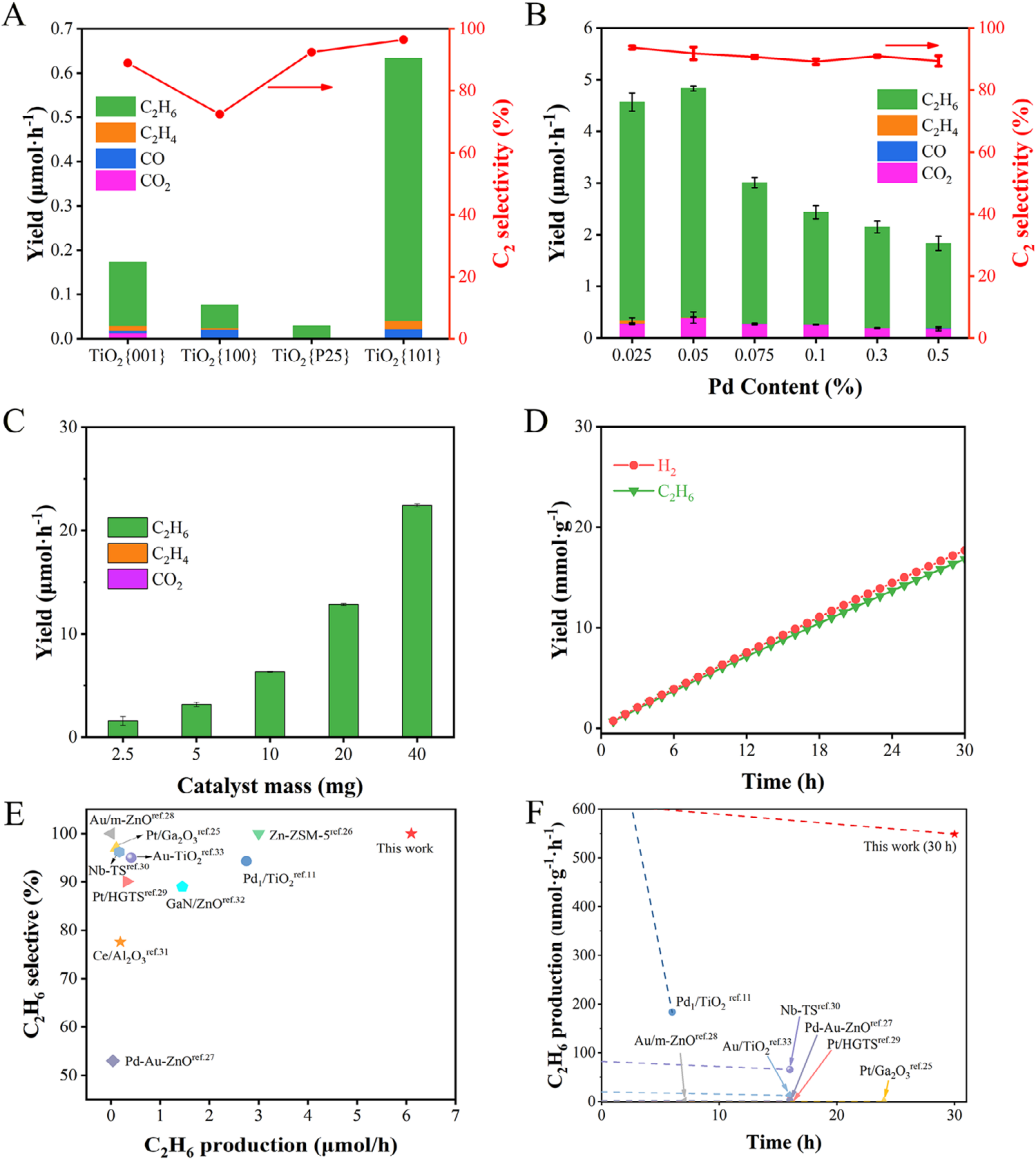
NOCM performance and the selectivity of the primary products (C_2_H_6_) over (A) TiO_2_{001}, TiO_2_{100}, TiO_2_{P25}, and TiO_2_{101}, (B) Pd_x_/TiO_2_{101} for Pd content optimization, (C) cal‐Pd_0.05_/TiO_2_{101} with different catalyst dosage. Reaction conditions: 10 mg of photocatalyst, CH_4_ flow rate of 10 mL/min, room temperature, 365 nm LED with 22.3 mW/cm^2^. (D) The temporal performance of the optimized cal‐Pd_0.05_/TiO_2_{101} photocatalyst. CH_4_ flow rate is 40 mL/min, and others are identical to previous studies. (E) Presentation of cal‐Pd_0.05_/TiO_2_{101} activity together with other representative photocatalysts in NOCM reaction. (F) Catalytic activity of cal‐Pd_0.05_/TiO_2_{101} vs. representative photocatalysts for NOCM (dashed line denotes the projected activity decay. The specific amount of catalyst is shown in Table S2.

TiO_2_ with exposed different facets was prepared by the hydrothermal method [16]. The photocatalytic activities of TiO_2_{001}, TiO_2_{100}, TiO_2_{101} and commercial TiO_2_ (P25) were primarily screened in a flow reactor (Figure S1A). Figure 1A shows the detected carbonaceous products of NOCM over TiO_2_{001}, TiO_2_{100}, TiO_2_{101} and P25, including C_2_H_6_, C_2_H_4_, CO and CO_2_. C_2_H_6_ and C_2_H_4_ are desirable products in the NOCM process, while CO and CO_2_ are overoxidized products caused by the O_L_ of TiO_2_ [17]. C_2_H_6_ is detected overall photocatalysts, suggesting that the excited TiO_2_ can break the C─H bonds of CH_4_ to form ·CH_3_, which then spontaneously couples to form C_2_H_6_ [11]. TiO_2_{101} exhibits a relatively higher C_2_H_6_ yield of 0.6 µmol·h^−1^ than TiO_2_{001} (0.18 µmol·h^−1^), TiO_2_{100} (0.06 µmol·h^−1^) and P25 (0.03µmol·h^−1^). Building on these observations, Figure S2 further compares facet‐engineered TiO_2_ samples with commercial P25, showing that although all exhibit activity in NOCM, their over‐oxidation behaviours differ significantly. Among them, TiO_2_{101} achieves the highest C_2_H_6_ yield with minimal CO_2_ formation, highlighting its superior balance between activity and suppression of overoxidation. The selectivity for C_2_H_6_ follows the order: TiO_2_{101} (96.5%) > P25 (92.4%) > TiO_2_{001} (88.9%) > TiO_2_{100} (72.4%). Thus, TiO_2_{101} is selected as the substrate. Pd single atoms and clusters were deposited on TiO_2_{101} by a photo‐deposition method [18].

The influence of Pd clusters was studied using a series of Pd_x_‐TiO_2_{101} catalysts (Figure 1B). All Pd decorated samples exhibit significantly higher C_2_H_6_ yield than pristine TiO_2_{101}. As the Pd content increases (quantified by ICP‐MS, see Table S1), the C_2_H_6_ yield follows a volcanic trend, rising from 4.2 µmol·h^−1^ over Pd_0.025_/TiO_2_{101} to a maximum of 4.5 µmol/h for Pd_0.05_/TiO_2_{101}, then declining at higher loadings. At the optimal loading, the catalyst achieves a balanced distribution of Pd single atoms and clusters. At higher Pd loading (>0.05 wt%), progressive aggregation into larger nanoparticles reduces the ratio of isolated Pd single‐atom sites to the Pd nanoclusters, thereby suppressing C_2_H_6_ yield and selectivity. All catalysts show nearly 90% selectivity. Previous studies have shown that high‐temperature steam treatment could effectively increase the grain boundary density of Pd catalyst, enhancing C─H bond activation [19]. Accordingly, Pd_0.05_/TiO_2_{101} was further treated with 450°C steam for 4 h. As shown in Figure S3, this pretreatment further improves C_2_H_6_ yield to 6 µmol/h while maintaining 90% selectivity.

Next, the effect of ethane synthesis on the catalyst amount was monitored. Figure 1C shows that the C_2_H_6_ yield over cal‐Pd_0.05_/TiO_2_{101} increases nearly linearly with catalyst amount under optimized reaction conditions, highlighting its strong scalability potential for larger‐scale applications. The effect of different CH_4_ flow rates on C_2_H_6_ yield over cal‐Pd_0.05_/TiO_2_{101} was investigated (Figure S1B). It is found that the C_2_H_6_ yield remains almost unchanged when the CH_4_ flow rate exceeds 10 mL/min. Notably, the C_2_H_6_ selectivity improves from 90 to nearly 100% when the CH_4_ flow rate increases to 40 mL/min. Product selectivity is dominated by the competition between C_2_H_6_ and CO_2_, with C_2_H_4_ detected only in trace amounts. Increasing the CH_4_ flow rate improves the mass transfer, shortening residence time, promoting rapid desorption of C_2_H_6_, and suppressing its over‐oxidation, thereby driving C_2_H_6_ selectivity close to 100%. This confirms reactant flow control as a key strategy for maximizing C_2_ selectivity in photocatalytic NOCM. Short residence times at high flow rates enhance mass transfer and limit the contact time between C_2_H_6_ and the catalyst surface. This reduced interaction can help suppress overreaction, thereby minimizing the formation of undesired byproducts [20, 21, 22, 23]. Control experiment results, as shown in Figure S4 suggest that both photocatalyst and light irradiation are indispensable for NOCM.

The stability of cal‐Pd_0.05_/TiO_2_{101} was investigated (Figure 1D). The primary product is C_2_H_6_, accompanied by trace amounts of C_2_H_4_. Throughout the 30‐hour testing period, the yields of H_2_ and C_2_H_6_ remain stable and closely align with the stoichiometric ratio, confirming that the reaction proceeds via NOCM. The absence of significant by‐products, such as excess H_2_ from carbon deposition, further supports the selective activation of C─H bond. In addition, the cycling stability test (Figure S5) further demonstrates that the photocatalyst maintains constant H_2_ and coupling product yields over five consecutive cycles, confirming reproducible and durable performance without site poisoning.

Representative results from previously reported photocatalytic NOCM systems are summarized in Figure 1E. The optimised cal‐Pd_0.05_/TiO_2_{101} exhibits a superior C_2_H_6_ yield exceeding 6 µmol·h^−1^, outperforming most reported photocatalysts by an order of magnitude, while maintaining an exceptionally high selectivity approaching 100%. Furthermore, catalyst stability, a major challenge in NOCM due to lattice oxygen consumption and coke formation [24], is benchmarked in Figure 1F. Most reported photocatalysts exhibit a significant decline in activity within 20 h, with losses ranging from 10%∼80%. In contrast, cal‐Pd_0.05_/TiO_2_{101} retains over 90% of its initial activity after 30 h of continuous operation. Its stable performance even extends to 50 h, yielding a total of 25 mmol_C2H6_/g_cat._, and a corresponding turnover number (TON) of 106. Overall, the photocatalyst presented in this study significantly surpasses existing NOCM systems that utilize CH_4_ as the sole reactant, in terms of selectivity, yield, and stability (Table S2) [11, 25, 26, 27, 28, 29, 30, 31, 32, 33].

The outstanding performance effectively surpasses the thermodynamic equilibrium limitation of the ethane yield at the given temperature (∼0.0004% at 310 K), by a factor of 115 [31]. Remarkably, the catalyst achieves a space time yield of 610 µmol·g^−1^·h^−1^ at room temperature, which is comparable to those thermocatalytic NOCM systems operating at elevated temperature (>623 K), while maintaining superior selectivity [34, 35].

**FIGURE 2:**
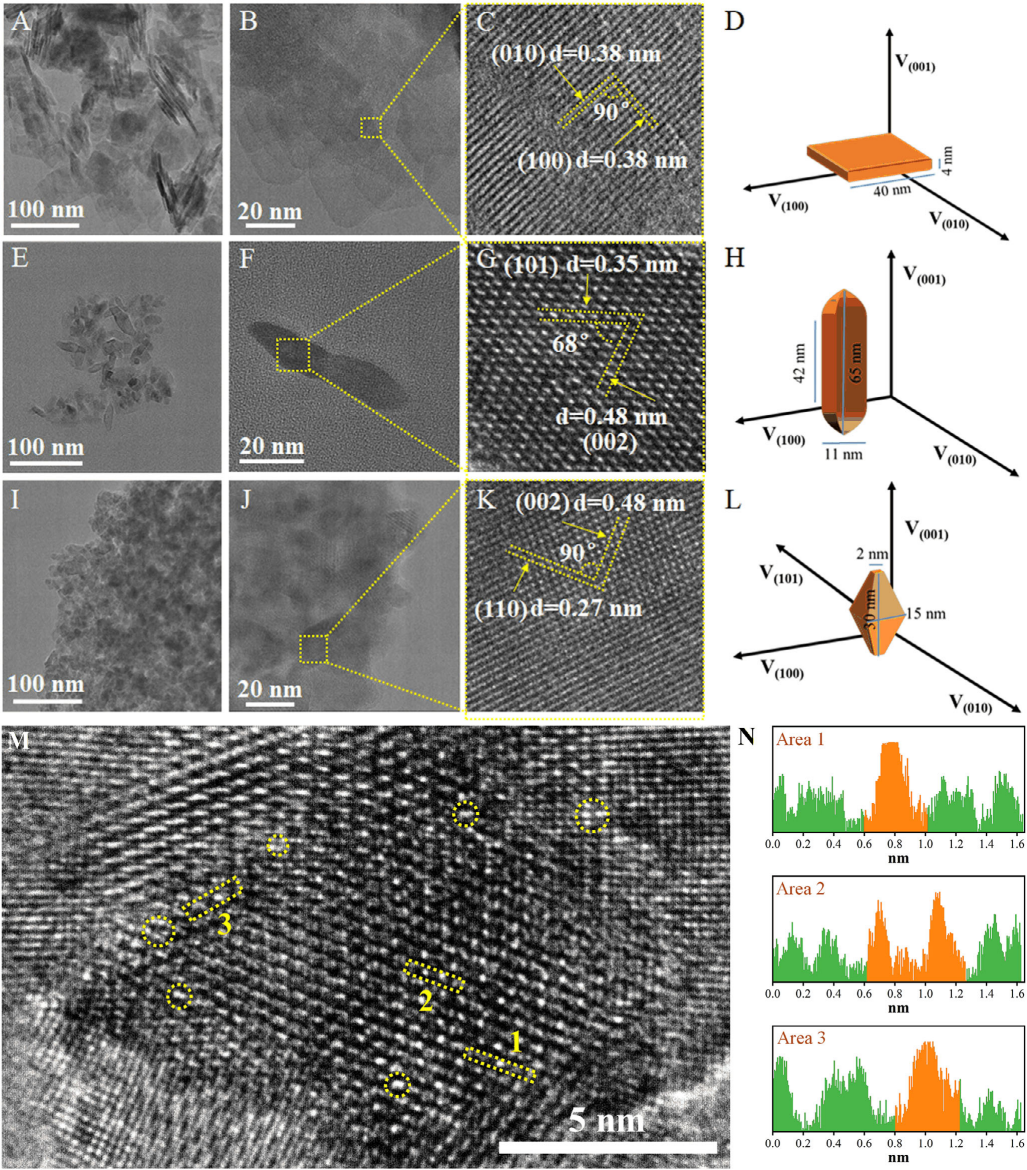
TEM images of TiO_2_{001} (A, B); TiO_2_{100} (E, F) and TiO_2_{101} (I, J); HRTEM images of **(C)** TiO_2_{001}, (G) TiO_2_{100} and (K) TiO_2_{101}, Texture coefficient plots of (D) TiO_2_{001}, (H) TiO_2_{100} and (L) TiO_2_{101}.(M) HAADF‐STEM images of cal‐Pd_0.05_/TiO_2_{101}, where Pd single atoms and clusters are marked by yellow dashed rectangle and cycle. (N) Line scan results of the area 1, area 2, and area 3 marked in (M).

The XRD patterns (Figure S6) show that all TiO_2_ samples with exposed facets exhibit a typical anatase TiO_2_ structure [36]. The morphologies of TiO_2_{001}, TiO_2_{100} and TiO_2_{101} are shown in Figure 2 and Figure S7. Figure 2A,D (corresponding to TiO_2_{001}), as well as Figure S7A show a rectangular sheet structure with a length of ∼40 nm, a width of ∼40 nm, and a height of 7 nm. Figure 2E,H (corresponding to TiO_2_{100}), along with Figure S7B, exhibits a columnar structure with a length of ∼50 nm. Figure 2I,L (corresponding to TiO_2_{101}) and Figure S7C display a truncated bipyramid structure with a length of ∼12 nm. All samples exhibit relatively uniform morphologies. The exposed facets of these TiO_2_ samples were determined based on the HRTEM images of TiO_2_{001} (Figure 2B,C), TiO_2_{100} (Figure 2F, G), and TiO_2_{101} (Figure 2J,K). As shown in Figure S8, all TiO_2_ samples with different exposed facets exhibit approximate BET specific surface areas of ∼100 m^2^/g, suggesting that specific surface area is not the main factor affecting photocatalytic activity. UV‐DRS measurements of the samples were conducted to evaluate their photo absorption abilities. The results are shown in Figure S9, presenting a similar photo absorption onset at ∼410 nm and a bandgap energy of 3.0 eV. In addition, VB‐XPS of TiO_2_{001}, TiO_2_{100}, and TiO_2_{101}) (Figure S10) were conducted to determine their detailed band structure. The results show that these three samples have similar valence band positions relative to the Fermi level. Thus, photo absorption ability and band structure are not the determining factors for the activity difference among these photocatalysts.

Then the Pd was deposited on the TiO_2_{101}. As shown in STEM images (Figure S11A,B), distinct differences in Pd dispersion are observed between Pd_0.05_/TiO_2_{101} and Pd_0.1_/TiO_2_{101}. Specifically, only a few scattered Pd clusters can be seen on Pd_0.05_/TiO_2_{101}, whereas Pd_0.1_/TiO_2_{101} exhibits a large number of dense Pd nanoparticles. This high particle density leads to obvious Pd aggregation, thus resulting in low atomic utilization efficiency of Pd. To clarify the existing forms of Pd species in cal‐Pd_0.05_/TiO_2_{101}, HAADF‐STEM images along with complementary line‐scan analysis were acquired (Figure 2M,N). As shown in Figure 2M, the TiO_2_{101} crystal planes are distinctly visible, along with points or clusters of varying brightness. Bright features in the atomic fringes, marked with yellow dashed rectangles and circles, were analyzed by line‐scan profiling. Due to the strong Z‐contrast of HAADF‐STEM, the enhanced intensity at these positions relative to the surrounding TiO_2_ lattice (Figure 2M, areas 1–3) is attributed to the higher atomic number of Pd (Z = 46) compared with Ti (Z = 22), confirming the coexistence of isolated Pd single atoms and sub‐nanometric clusters [19]. The Pd modification, followed by steam treatment, did not change the structure of the TiO_2_{101} substrate (Figure S12).

Further identification of Pd species was achieved by CO adsorption DRIFTS and temperature‐programmed CO desorption. As shown in Figure S13, CO‐DRIFTS spectra collected from 1 to 5 min under continuous CO flow show progressive growth of absorption bands, reaching near‐saturation by 5 min. The band at ∼2100 cm^−1^ corresponds to CO linearly adsorbed on oxidized Pd single atoms (Pd^2+^/Pd^+^), while the band at ∼1920 cm^−1^, highlighted in the insert, corresponds to bridging CO on metallic Pd clusters, requiring adjacent Pd atoms. As shown in Figure S14, temperature‐programmed CO desorption further distinguishes the binding strength: the ∼2100 cm^−1^ band decreases more rapidly with increasing temperature than the ∼1920 cm^−1^ band. This differential desorption behaviour indicates weaker CO adsorption on isolated Pd single atoms and stronger adsorption on Pd clusters, providing clear evidence for the coexistence of distinct Pd species.

The crystal structures of Pd_x_‐TiO_2_{101} and cal‐Pd_x_/TiO_2_{101} samples are shown in XRD patterns (Figure S15). After the introduction of Pd, the standard XRD patterns of TiO_2_{101} remained unchanged, indicating a stable framework. No diffraction peaks assignable to Pd‐related phases are observed, most likely due to the low Pd loading and high dispersion. Raman spectroscopy provides a means to confirm the structures of TiO_2_{101} and cal‐Pd_0.05_/TiO_2_{101} [37]. The molecular vibrations of TiO_2_ with exposed different facets normally exhibit different intensities. As shown in Figure S16, the weakened B_1g_ and A_1g_ modes at 397 cm^−1^ and 511 cm^−1^ indicate minimal {001} facet exposure in TiO_2_{101}, which is consistent with its truncated bipyramid structure. In contrast, the higher intensity of the E_g_ mode at 145 cm^−1^ indicates preferential {101} facet exposure in TiO_2_{101}. After Pd was deposited on TiO_2_{101}, two characteristic changes of the E_g_ mode at 145 cm^−1^ are evident: decreased intensity and a shift to lower frequencies. It is concluded that Pd clusters deposited on the {101} facet affect the surface Ti─O bonds [38]. UV‐DRS measurements of Pd_x_/TiO_2_{101} and cal‐Pd_0.05_/TiO_2_{101} were conducted to evaluate their photo absorption abilities. The results are shown in Figure S17; all these photocatalysts exhibited a similar photo absorption onset at ∼410 nm and a bandgap energy range of 2.97–3.05 eV. This illustrates that the deposition of Pd clusters has little effect on photo absorption ability or bandgap energy.

Charge separation plays a key role in significantly influencing the photocatalytic process. Steady‐state PL spectroscopy was used to investigate the charge separation efficiency of TiO_2_{001}, TiO_2_{101}, Pd_0.05_‐TiO_2_{101}, and TiO_2_{101} (Figure S18). TiO_2_{001} and TiO_2_{101} exhibit a relatively similar PL emission peak intensity at 475 nm, while TiO_2_{100} shows the highest PL intensity. This suggests that TiO_2_{100} has more severe charge recombination than TiO_2_{001} and TiO_2_{101} [39]. Time‐resolved PL decay measurements were conducted on TiO_2_{001}, TiO_2_{100}, and TiO_2_{101} to further illustrate charge separation from the time domain (Figure S19). Compared with TiO_2_{100} (where the decay lifetime of photogenerated charges is 2.6 ns), the photogenerated charges on the TiO_2_{001} (3.6 ns) and TiO_2_{101} (3.7 ns) have a longer lifetime, which is more conducive to their participation in the photocatalytic reaction [40]. In addition, it is known that photogenerated electrons preferentially migrate to the {101} facets [41, 42]. Photo‐generated electrons on the {101} facet are shallowly confined at the five‐coordinated Ti atoms (Ti_5c_) located on the surface. In sharp contrast, the photogenerated electrons on the {001} facet are deeply trapped at the six‐coordinated Ti atoms (Ti_6c_) situated in the subsurface region. Consequently, for TiO_2_{001}, the bulk transfer of charges becomes much more difficult. This impeded charge bulk transfer process significantly promotes the recombination of photogenerated electron‐hole pairs, ultimately resulting in a shorter carrier lifetime compared to TiO_2_{101} [43].

**FIGURE 3:**
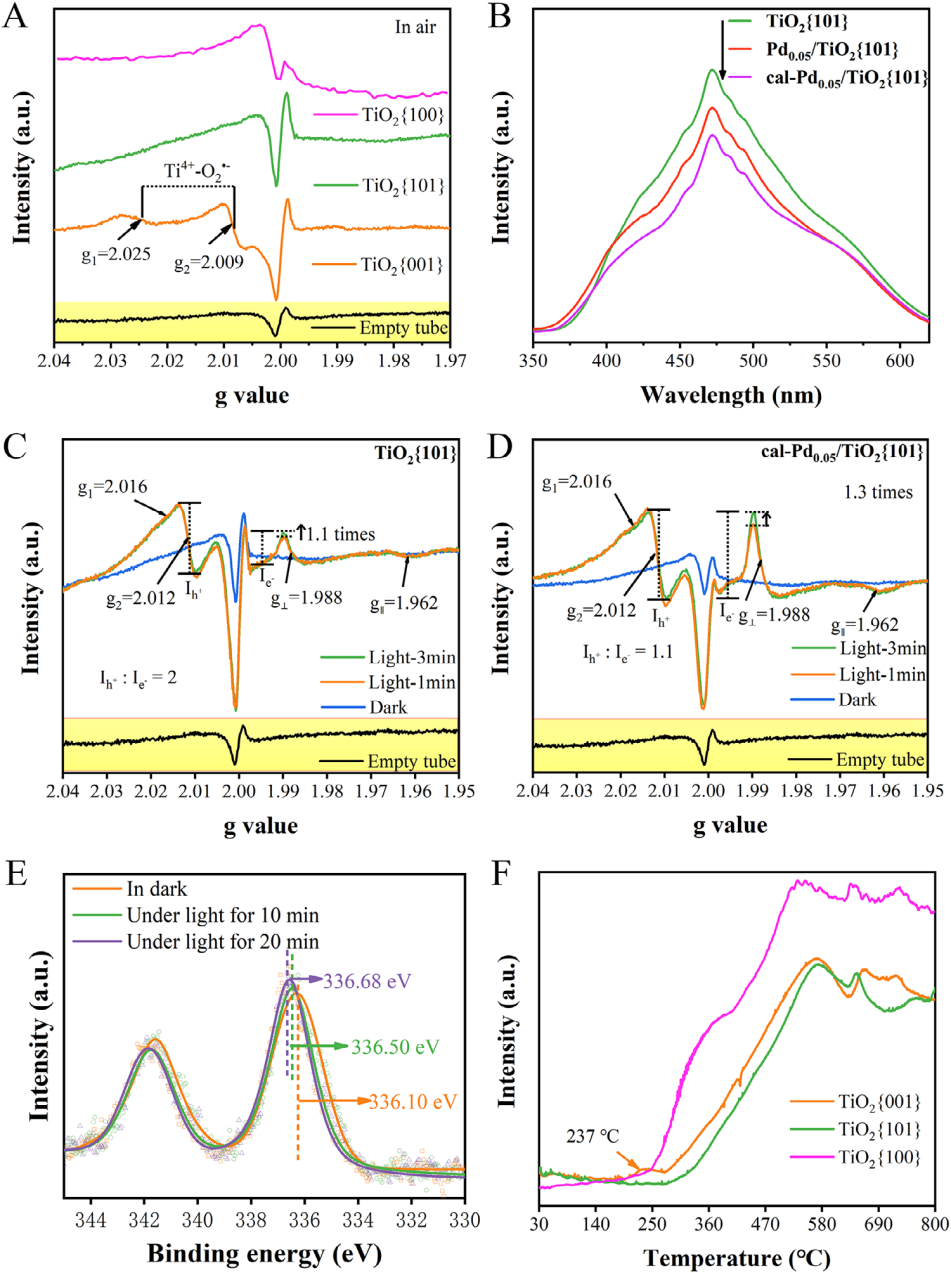
(A) EPR spectra of TiO_2_{100}, TiO_2_{101} and TiO_2_{001}. (B) Fluorescence spectra of TiO_2_{101}, Pd_0.05_/TiO_2_{101} and cal‐Pd_0.05_/TiO_2_{101}. In situ EPR spectra of (C) TiO_2_{101} and (D) cal‐Pd_0.05_/TiO_2_{101} in the dark and under light irradiation. (E) In situ Pd 3d XPS spectra of cal‐Pd_0.9_/TiO_2_{101} in dark and under light irradiation. (F) H_2_‐TPR of TiO_2_{001}, TiO_2_{100} and TiO_2_{101}.

EPR experiments of TiO_2_{001}, TiO_2_{100}, and TiO_2_{101} were further conducted at 100 K to investigate the surface properties. As shown in Figure 3A, the signals at g_1_ = 2.025 and g_2 =_ 2.009 for TiO_2_{001} in the dark are assigned to Ti^4+^‐O^2−^, which results from dissociatively adsorbed O_2_ [44]. Notably, no obvious EPR signals are observed for TiO_2_{100} and TiO_2_{101}. The above results suggest that TiO_2_{001} preferentially adsorbs O_2_, which might increase its electron transfer ability. The CH_4_ conversion reaction over TiO_2_{001}, TiO_2_{100} and TiO_2_{101} at a CH_4_/O_2_ ratio of 100:1 was conducted. As shown in Figure S20, TiO_2_{001} exhibits higher CH_4_ conversion performance, but CO_2_ and CO are detected as the main products.

After modification with Pd clusters, transient photocurrent (I–T) measurements provide direct evidence of charge‐carrier behavior. The photocurrent densities follow the order cal‐Pd_0.05_/TiO_2_{101} > Pd_0.05_/TiO_2_{101} > TiO_2_{101} (Figure S21). Since all three samples show similar UV–vis absorption thresholds and band gaps (∼3.0 eV), the difference arises from charge separation efficiency rather than light absorption. The PL spectra are shown in Figure 3B. The emission peak intensity of Pd_0.05_‐TiO_2_{101} is weakened, suggesting that charge recombination is suppressed. Steam pretreatment further improves charge separation. In situ EPR experiments were used to study the direction of charge transfer. As shown in Figure 3C, the EPR signals of TiO_2_{101} at g_⊥_ = 1.988 and g_∥_ = 1.988 under light irradiation are categorized as Ti^3+^ species, which arise from trapped photo‐induced electrons. The signals of TiO_2_{101} at g_1_ = 2.016 and g_2_ = 2.012 arise from trapped photo‐induced holes [45, 46]. The intensity of the Ti^3+^ signal at g_⊥_ = 1.988 increases by 1.1 times when light irradiation is extended from 1 min to 3 min. Similar EPR signals were also observed for cal‐Pd_0.05_/TiO_2_{101} under light irradiation (Figure 3D). However, the Ti^3+^ signal at g_⊥_ = 1.988 for cal‐Pd_0.05_/TiO_2_{101} exhibits a significant intensity enhancement by 1.3 times. This may be due to modified Pd single atoms and clusters acting as hole acceptors, which further leads to an increased electron accumulation on titanium as Ti^3+^ [47]. In addition, the ratio of the signal at g_2_ = 2.012 to that at g_⊥_ = 1.988 for cal‐Pd_0.05_/TiO_2_{101} is lower than that for TiO_2_{101}. The above results further indicate that the hole concentration is decreased, likely due to the hole‐trapping effect of Pd clusters.

High‐resolution Ti 2p and O 1s XPS spectra of cal‐Pd_0.05_/TiO_2_{101} (Figures S22 and S23) confirm that Pd^2+^ species are stabilized through coordination with lattice oxygen atoms. In situ XPS was employed to consolidate the migration behavior of photogenerated charge under illumination and the role of Pd in this process. However, cal‐Pd_0.05_/TiO_2_{101} exhibits a very weak Pd signal (Figure S24), which is attributed to the extremely low Pd loading and the limited detection sensitivity of the in situ XPS spectra (Thermo ESCALAB 250Xi instrument). PL (Figure S25) and transient photocurrent measurements (Figure S26) confirm that Pd_0.9_/TiO_2_{101} and cal‐Pd_0.05_/TiO_2_{101} follow the same electron‐transfer mechanism. The consistency of their spectral profiles validates Pd_0.9_/TiO_2_{101} as a suitable substitute for mechanistic studies using in situ XPS. Thus, cal‐Pd_0.9_‐TiO_2_{101}, prepared via the same procedure but with a higher Pd content, was used to identify the function of Pd species [48]. As shown in Figure 3E, the Pd 3d XPS peak of cal‐Pd_0.9_/TiO_2_{101} centers at ∼336.10 eV, which is close to the binding energy of Pd^2+^ (336.55 eV) [49]. Notably, the Pd 3d XPS spectra show that the binding energy of Pd 3d_5/2_ shifts from 336.10 eV (in the dark) to 336.50 eV after 10 min of light irradiation and to 336.68 eV after 20 min of light irradiation. The upshift of the Pd 3d_5/2_ binding energy under light irradiation confirms that Pd species act as hole acceptors.

Then, CH_4_ temperature‐programmed desorption (CH_4_‐TPD) experiments were performed to evaluate the interactions between the photocatalysts and CH_4_ (Figure S27). All samples exhibit a single CH_4_ desorption peak, centered at 100°C, 125°C and 150°C for TiO_2_{100}, TiO_2_{001} and TiO_2_{101}, respectively. This result indicates that CH_4_ has a stronger interaction with TiO_2_{101} than with TiO_2_{100} and TiO_2_{001}, which benefits the NOCM reaction. In addition, the CH_4_ conversion products over TiO_2_ with exposed different facets (Figure 1A) suggest that O_L_ slightly participates in CH_4_ conversion, which leads to the over‐oxidation of desired products to CO and CO_2_. Such oxidation by O_L_ is accompanied by the consumption of O_L_ and the formation of oxygen vacancies (O_V_), which will inevitably affect the activity and stability of the catalyst [11].

Therefore, H_2_ temperature‐programmed reduction (H_2_‐TPR) experiments were carried out to study the stability of O_L_ in the catalysts. As shown in Figure 3F, TiO_2_{001}, TiO_2_{100}, and TiO_2_{101} exhibit a broad reduction feature with a similar range, centered at 571°C. Notably, the onset of the reduction peak for TiO_2_ {001} occurred at approximately 237°C, indicating that the O_L_ in TiO_2_{001} has higher oxidizing ability compared with that in TiO_2_{101} and TiO_2_{100}, and its O_L_ can be readily reduced [50]. The onset of the reduction peak for TiO_2_{101} and TiO_2_{100} occurs later than that for TiO_2_{001}. Therefore, the low selectivity of TiO_2_{001} under oxygen‐lean conditions may be attributed to its surface two‐fold coordinated O (O_2c_) atoms—these O_2c_ atoms possess higher oxidizing capacity and thus actively participate in CH_4_ conversion. In contrast, TiO_2_{101} has a surface composed of 50% three‐fold coordinated O (O_3c_) atoms and 50% O_2c_ atoms. The key distinction lies in the fact that the surface O atoms of TiO_2_{101} exhibit significantly weaker oxidizing capacity; this low oxidizing capacity renders them relatively stable, such that they rarely participate in CH_4_ conversion or induce overoxidation [51, 52]. This characteristic of TiO_2_{101} stands in contrast to TiO_2_{001} (which has 100% O_2c_ atoms with strong oxidizing capacity) and is well consistent with the Raman and XPS results.

As shown in Figure S28, both TiO_2_{101} and TiO_2_{001} display the characteristic Raman modes of anatase TiO_2_, including the dominant Eg(1) mode at ∼144 cm^−1^ (symmetric O–Ti–O bending), the B_1g_(1) mode at ∼399 cm^−1^ (antisymmetric O–Ti–O bending), and the B_1g_(2) mode at ∼519 cm^−1^ (antisymmetric Ti–O stretching coupled with O–Ti–O bending). Distinct spectral features in the higher‐wavenumber region reveal differences in surface oxygen coordination. At ∼612 cm^−1^ and ∼638 cm^−1^, both peaks are assigned to vibrational modes dominated by O_3c_ stretching, with the former corresponding to surface O_3c_ sites and the latter to bulk O_3c_ sites. TiO_2_{101} exhibits a noticeably stronger relative intensity at 612 cm^−1^ compared to TiO_2_{001}, confirming that surface O_3c_ sites are more abundant on the {101} facet. This is consistent with the well‐established structural feature of the anatase (101) surface, which is predominantly terminated by O_3c_ sites within its characteristic sawtooth geometry. In contrast, at ∼720 cm^−1^, TiO_2_{101} exhibits weaker intensity than TiO_2_{001} due to Ti–O_2c_ stretching, reflecting the lower density of exposed O_2c_ sites on the {101} facet compared with the {001} facet, where O_2c_‐terminated structures are more prevalent.

These Raman findings are corroborated by high‐resolution O1s XPS analysis (Figure S29). The dominant peak at ∼529.7 eV (green component) corresponds to bulk lattice oxygen in a stable Ti–O–Ti environment, consistent with O_3c_ sites in the anatase lattice. The component at ∼531.6 eV (blue component) is attributed to surface oxygen species in lower‐coordination environments, including O_2c_ and hydroxylated bridging sites. The two O1s spectra exhibit distinct deconvolution profiles that reflect different surface oxygen coordination. For TiO_2_{101}, the spectrum is dominated by the sharp ∼529.7 eV peak with only a minor high‐binding‐energy shoulder, indicating that most oxygen species occupy stable, high‐coordination sites. The relatively small ∼531.6 eV contribution agrees with the intrinsically low density of under‐coordinated oxygen on the {101} facet. In contrast, TiO_2_{001} shows a substantially larger ∼531.6 eV component, revealing a higher population of under‐coordinated surface oxygen species.

**FIGURE 4:**
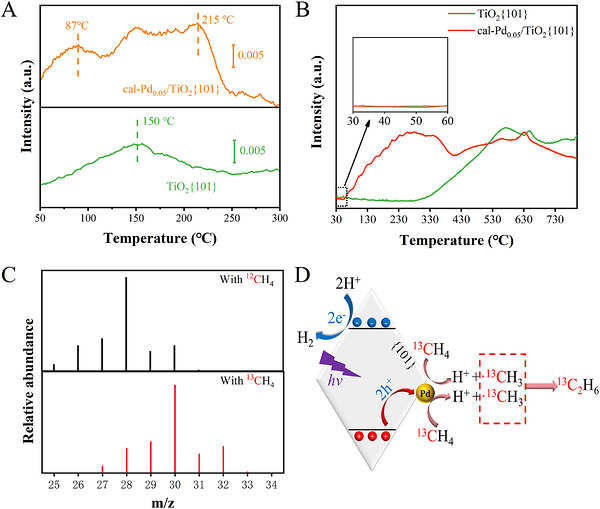
(A) CH_4_ TPD and (B) H_2_‐TPR profiles of TiO_2_{101}and cal‐Pd_0.05_/TiO_2_{101}. (C) Mass spectra of the isotope labelling experiments. (D) Mechanism of NCOM over cal‐Pd_0.05_/TiO_2_{101}.

Subsequently, the surface chemical properties following Pd incorporation on TiO_2_{101} were investigated, as shown in Figure 4. In the CH_4_‐TPD experiments (Figure 4A), pristine TiO_2_{101} displays a single CH_4_ desorption peak centered at 150°C. In contrast, the TPD profile of cal‐Pd_0.05_/TiO_2_{101} is significantly broadened and exhibits three distinct CH_4_ desorption peaks. The newly emerged peaks at 87°C and 215°C are attributed to CH_4_ adsorption on Pd clusters [53]. The presence of a higher‐temperature desorption peak indicates that Pd modification substantially enhances the adsorption interaction between the catalyst surface and CH_4_ [54].

The enhanced kinetics of charge transfer and surface reaction are evidenced by the calculated apparent quantum efficiency (AQE). Specifically, cal‐Pd_0.05_/TiO_2_{101} achieves an AQE of 0.49%. Notably, compared to pristine TiO_2_{101} without Pd modification, the AQE of cal‐Pd_0.05_/TiO_2_{101} is enhanced by a factor of 10. This substantial improvement is attributed to the synergistic interaction between the Pd active sites and the {101} crystal facet of TiO_2_, which facilitates efficient separation of photogenerated electron‐hole pairs and promotes the activation of CH_4_ molecules.

Considering the outstanding activity of this catalyst, a detailed investigation of the optimised catalyst was conducted to elucidate the underlying mechanisms. H_2_ temperature‐programmed reduction (H_2_‐TPR) experiments were carried out. As shown in Figure 4B, the reduction peak of cal‐Pd_0.05_/TiO_2_{101} is shifted to a lower temperature compared to that of TiO_2_{101}; more notably, the onset (starting position) of its reduction peak is shifted substantially toward lower temperatures relative to TiO_2_{101}. These observations together indicate enhanced oxidizability of cal‐Pd_0.05_/TiO_2_{101}. Additionally, as shown in the inset, there is no peak in the range of 30–60°C, a peak that is typically associated with the reduction of PdO [55, 56]. This indicates that the Pd species loaded on TiO_2_ exist as clusters instead of PdO. Subsequently, the selectivity is improved by shortening the reactant contact time at the interface at a faster flow rate, which not only boosts the external mass transfer rate but also limits the contact of C_2_H_6_ with the catalyst's active sites. Such limited contact effectively mitigates the occurrence of undesired side reactions, thereby contributing to the increased selectivity [22, 23]. Afterward, Raman spectroscopy and FT‐IR spectroscopy were employed to assess potential carbonaceous deposition. As shown Figures S30 and S31, no significant peaks corresponding to the characteristic peaks of alkanes and olefins (at 1165 cm^−1^, 1240 cm^−1^, 1385 cm^−1^, and 1470 cm^−1^) are observed during the 1 h to 5 h of reaction, indicating no carbon deposition [57]. FT‐IR spectra of fresh cal‐Pd_0.05_/TiO_2_{101} and the sample after 5 h of CH_4_ oxidation were recorded (Figure S32). No obvious signals of oxygen‐containing organic compounds are observed, suggesting that CH_4_ conversion proceeds via dehydrogenated coupling under oxygen‐lean conditions. Additional post‐reaction characterizations further confirm the robustness of the catalyst: N_2_ adsorption–desorption (Figure S33) shows preserved mesoporous structure, XRD patterns (Figure S34) reveal unchanged crystalline framework, and UV–vis DRS spectra (Figure S35) indicate stable optical properties. Isotope‐labeling experiments were conducted in a batch reactor to confirm the carbon source of the produced C_2_H_6_ (Figure 4C). The cal‐Pd_0.05_/TiO_2_{101} was reacted with ^13^CH_4_ under light irradiation. The m/z of dominant peaks attributed to C_2_H_6_ increases by 2 [7]. This proves that CH_4_ is indeed the carbon source for C_2_H_6_ formation. Beyond confirming the carbon source, in situ spectroscopic evidence further substantiates the reaction pathway. As shown in Figure S36, in situ DRIFTS measurements on cal‐Pd_0.05_/TiO_2_{101} under a CH_4_ atmosphere reveal a distinct band at 1374 cm^−1^ that increases progressively with visible‐light irradiation, corresponding to the symmetric C–H deformation vibration of surface‐adsorbed CH_3_^*^.

Based on the above results, a mechanism for NOCM over cal‐Pd_0.05_/TiO_2_{101} is proposed (Figure 4D). Under light irradiation, the excited photoelectrons accumulate on the {101} facet of TiO_2_, Pd single atoms and clusters act synergistically to enhance hole transfer, promote CH_4_ C–H activation, and generate CH_3_^*^. Subsequently, H^+^ is reduced to H_2_ by the accumulated photoelectrons at the {101} facet [58], and CH_3_^*^ couples to form C_2_H_6_.

## 3 Conclusion

In summary, we successfully synthesized a Pd cluster‐modified TiO_2_ photocatalyst with strategically exposed {101} facets and employed it for the nonoxidative coupling of methane (NOCM) in a continuous flow reactor. Under 365 nm light irradiation, using CH_4_ as the sole reactant, the system achieves a high C_2_H_6_ production rate of 600 µmol·h^−1^·g^−1^ at ambient temperature—a performance metric comparable to traditional thermocatalytic processes operating above 400°C. Remarkably, the catalyst demonstrates near 100% selectivity towards C_2_ hydrocarbons and maintains stable performance for over 30 h, exceeding the limitations of prior systems and achieving a turnover number (TON) of 106.

An integrated suite of characterization techniques elucidates the origin of this enhanced activity and stability. Photophysical measurements confirm that the {101} facet intrinsically promotes efficient charge separation. In situ XPS and EPR analyses demonstrate that the decorated Pd single atoms and clusters act as effective hole acceptors, facilitating charge transfer, while CH_4_‐TPD profiles reveal these species significantly strengthen methane adsorption on the catalyst surface. The synergy between the {101} facet and Pd single atoms and clusters efficiently promotes the dissociation of adsorbed CH_4_ into H^+^ and CH_3_^*^. Subsequently, H^+^ is reduced to H_2_ at the facet, and CH_3_^*^ radicals undergo coupling to form ethane (C_2_H_6_). Furthermore, H_2_‐TPR experiments prove that the three‐fold coordinated oxygen atoms on the stable {101} facet exhibit greater resistance to reduction compared to other facets. This inherent stability crucially suppresses the involvement of lattice oxygen, thereby preventing overoxidation and ensuring the high selectivity and long‐term durability of the NOCM reaction. Overall, this study provides fundamental insights and a precise design strategy for constructing robust photocatalysts to achieve efficient and selective methane valorization under mild conditions.

## 4 Experimental Section

### 4.1 Chemicals and Materials

Deionized water was purchased without further purification. Titanium(IV) butoxide (C_16_H_36_O_4_Ti, 98.5%, Greagent), hydrofluoric acid (HF, 40%, Greagent), titanium(IV) chloride (TiCl_4_, 99%, Greagent), hydrochloric acid (HCl, 37%, Adamas), ammonium hydroxide solution (NH_4_OH, 29%, Adamas), ammonium sulfate ((NH_4_)_2_SO_4_, 99.5%, Greagent), ammonium chloride (NH_4_Cl, 99%, Adamas) and potassium tetrachloropalladate(II) (K_2_PdCl_4_, 99%, Adamas) were used in the experimental studies.

### 4.2 Preparation of Facet Exposure TiO_2_

All TiO_2_ samples were prepared according to the previous study.

**TiO_2_{001}**: Typically, 25 mL of C_16_H_36_O_4_Ti and 3 mL of HF were mixed and transferred into a Teflon‐lined autoclave, followed by heating at 180°C for 96 h. The collected product was centrifuged and washed several times with H_2_O and ethanol. Finally, the white powder was dried at 80°C for 12 h.

**TiO_2_{100} and TiO_2_{101}**: A total of 6.6 mL of TiCl_4_ and 7 mL of HCl were mixed into 14 mL of H_2_O, followed by stirring at 0°C for 1 h. 50 mL of NH_4_OH (5.5 wt%) was added dropwise to the mixture at room temperature. Then, the pH of the above mixture was adjusted to 6 using NH_4_OH (4 wt%), and the mixture was stirred at room temperature for 2 h. The collected white powder was centrifuged and washed several times with H_2_O and ethanol. Then, 2.0 g of dried white powder and 0.5 g of (NH_4_)_2_SO_4_ (for synthesizing TiO_2_{100}) or 0.2 g of NH_4_Cl (for synthesizing TiO_2_{101}) were dissolved in 30 mL of ethanol and water mixture (1:1). After stirring for 1 h, the solution was transferred into a Teflon‐lined autoclave, followed by heating at 180°C for 24 h. The collected white powder was centrifuged and washed several times with H_2_O and ethanol. Finally, the white powder was dried at 80°C for 12 h.

**Pd_x_/TiO_2_{101}**: A total of 0.25 g of TiO_2_ was first suspended in the mixture of methanol and water (1:9). A certain amount of K_2_PdCl_4_ was added, and the suspension was purged with ultrapure Ar for 30 min under stirring in the dark. Then the suspension was sealed and irradiated for 4 h. The collected product was centrifuged and washed several times with H_2_O and ethanol. Finally, the white powder was dried at 80°C for 12 h and designed as Pd_x_/TiO_2_, where the actual Pd loadings were measured by ICP‐AES (*x = 0.025, 0.05, 0.075, 0.1, 0.3, 0.5)*.

**cal‐Pd_0.05_/TiO_2_{101}**: A total of 0.2 g of Pd_0.05_‐TiO_2_{101} was treated at 450°C for 0.5 h under Ar flow with steam (Ar flow rate: 25 mL·min^−1^; the saturator contained water at 25°C).

### 4.3 Characterization

XRD patterns were collected using a Bruker D8 Advance diffractometer equipped with a solid‐state X’ Celerator detector (power: 2.2 kW) and Cu Kα radiation, over a 2θ range from 5 to 80°. VB‐XPS measurements were performed using a Thermo Scientific K‐Alpha system with an Al Kα X‐ray source. In situ XPS measurements were performed using a Thermo Scientific ESCALAB 250Xi system with an Al Kα X‐ray source; a 365 nm LED light was used as the excitation source. PL spectra were recorded using a Hitachi F4500 spectrofluorometer. EPR measurements were conducted on a Bruker E500‐9.5/12 spectrometer operating at X‐band frequency and 100 K, with 365 nm LED used as the irradiation source. CH_4_‐TPD and H_2_‐TPR analyses were performed using an automated chemisorption analyzer (Micromeritics AutoChem II 2920). TEM images were obtained using a FEI Talos F200X microscope equipped with an Energy‐dispersive X‐ray (EDX) Detector. SEM images were acquired using a Phenom Pharos microscope. The UV–vis absorption spectra (UV–vis abs) of the samples were recorded using a Shimadzu UV‐3600 plus spectrophotometer, with BaSO_4_ as the reference standard. Time‐resolved PL decay spectra were collected using an Edinburgh Instruments FLSP920 spectrofluorometer at room temperature (excitation wavelength: 310 nm). Raman spectra were obtained using a Renishaw Invia Basis Raman microscope.

### 4.4 Isotope Labeling Experiment

The reactor was filled with ^13^CH_4_ and irradiated for 1 h. The collected products were then analyzed using a GC‐MS (QP2010Plus, Shimadzu Co., Ltd).

### 4.5 Photocatalytic Activities for CH_4_ Conversion

10 mg of the sample powder was suspended in 10 mL of H_2_O and filtered through a nylon membrane (diameter: 35 mm). The uniform film was then dried at 60°C for 12 h. The film was placed in the flow reactor with a temperature probe to monitor the reaction temperature. First, the system was purged with Ar (50 mL·min^−1^) for 30 min. Second, the reactor was irradiated by a 365 nm LED light source with a CH_4_ flow rate of 10 mL/min. Then the outlet gases were detected by an SP‐3420A GC equipped with a thermal conductivity detector (TCD) and a flame ionization detector (FID). The CH_4_ flow rate over the control experiment was 10 mL/min, 20 mL/min, 30 mL/min, and 40 mL/min. The CH_4_ flow rate in the stability experiment was 40 mL/min.

### 4.6 Calculation of Selectivity

The selectivity of C_2_H_6_ was calculated based on the observable products, as shown below:

$$C_{2}H_{6}\;\mathrm{selectivity}\;=\frac{n\left(C_{2}H_{6}\right)}{n\left(C_{x}H_{y}\right)+n\left(CO_{2}\right)+n\left(CO\right)}\times 100\%$$

In the above equation, n represents the amount of substance of each molecule, C_x_H_y_ represents all C_2_ hydrocarbons that can be detected.

Turnover number (TON) was calculated by the following equation:

$$TON=\frac{2\times moles\;of\;C_{2}H_{6}\;yield}{moles\;of\;Pd\;atoms}$$

## Conflicts of Interest

The authors declare no conflict of interest.

## Supporting information




**Supporting File 1**: advs76511‐sup‐0001‐SuppMat.docx.

## Data Availability

The data that support the findings of this study are available from the corresponding author upon reasonable request.
