# Supplementary material for: Synergy of Pd Clusters and TiO2 {101} for Photocatalytic Nonoxidative Coupling of Methane
Source: Adv Sci (Weinh). 2026 Jul 13;13(55):e24322. doi: 10.1002/advs.202524322 (PMC13360133; doi:10.1002/advs.202524322)
Supplement: Supplementary file 1 [file ADVS-13-e24322-s001.docx]

**Supplementary Information**

**Synergy of Pd Clusters and TiO_2_ {101}** **for Photocatalytic Nonoxidative Coupling of Methane**

Jianlong Yang^1,2^, Annika S Tang^3,^ and Junwang Tang^1^ *

^1^Industrial Catalysis Center, Department of Chemical Engineering, Tsinghua University, Beijing 100084, China.

^2^ Yulin Innovation Institute of Clean Energy, Yulin, 719000, China

^3^ Department of Engineering Science, University of Oxford, Oxford OX1 3PJ, United Kingdom

** Corresponding author, e-mail address:* *jwtang@tsinghua.edu.cn*

Figure S1. (A) Detailed configuration of flow photocatalytic reactor system. 365 nm LED was used as light source and 10 mg of photocatalyst particles were evenly distributed over the film of 10 cm^2^. (B) NOCM performance of cal-Pd_0.05_/TiO_2_{101} with difference catalyst dosage.

Figure S2. Over-oxidation products (CO, CO_2_) over TiO_2_{001}, TiO_2_{100}, TiO_2_{P25} and TiO_2_{101}

As shown in Figure S2, all three facet-engineered TiO_2_ samples exhibit higher overall activity than commercial TiO_2_{P25}, confirming the benefit of facet engineering. However, their over-oxidation behaviors differ significantly. TiO_2_{001} shows the highest CO_2_ yield of 0.013 μmol·h^-1^ and a CO yield of ~0.006 μmol·h^-1^, indicating severe over-oxidation. In contrast, TiO_2_{100} and TiO_2_{101} produce much higher CO yields (~0.021 and ~0.022 μmol·h^-1^, respectively), while their CO_2_ signals remain below the detection limit of our GC system (20 ppm).

Figure S3. NOCM performance and the selectivity of the primary products (C_2_H_6_) over cal**-**Pd_0.05_/TiO_2_{101}. Reaction conditions: 10 mg of photocatalyst, CH_4_ flow rate of 10 mL/min, room temperature, 365 nm LED with 22.3 mW/cm^2^.

Figure S4. Control experiments for NOCM over cal-Pd_0.05_/TiO_2_{101}

Figure S5. Cycling stability test of the photocatalyst over 5 cycles. Reaction conditions: 10 mg of photocatalyst, CH_4_ flow rate of 10 mL/min, and 365 nm LED with 22.3 mW/cm^2^.

Each cycle lasted 4 hours with periodic sampling at 1-hour intervals, and the atmosphere was purged with N_2_ between cycles before reintroducing CH_4_. The production rates of H_2_ and coupling products remained constant across all cycles, confirming high reproducibility and stable photocatalytic performance without site poisoning.

Figure S6. XRD patterns of TiO_2_{001}, TiO_2_{100} and TiO_2_{101}.

Figure S7. SEM images of (A) TiO_2_{001}, (B) TiO_2_{100} and (C) TiO_2_{101}.

Figure S8. BET analysis of (A) TiO_2_{001}, (B) TiO_2_{100} and (C) TiO_2_{101}.

Figure S9. (A) UV-DRS spectra and related (B) Tauc plot of TiO_2_{001}, TiO_2_{100} and TiO_2_{101}.

Figure S10. VB-XPS patterns of TiO_2_{001}, TiO_2_{100} and TiO_2_{101}

Figure S11. STEM image of (A) Pd_0.05_/TiO_2_{101} and (B) Pd_0.1_/TiO_2_{101}.

Figure S12. SEM image of cal-Pd_0.05_/TiO_2_{101}.

Figure S13. CO adsorption DRIFTS spectra of cal-Pd_0.05_/TiO_2_{101}

Figure S14. Temperature-programmed CO desorption DRIFTS spectra of cal-Pd_0.05_/TiO_2_{101}

Figure S15. XRD patterns of Pd_0.025_-TiO_2_{101}, Pd_0.05_-TiO_2_{101}, Pd_0.075_-TiO_2_{101}, Pd_0.1_-TiO_2_{101}, Pd_0.3_-TiO_2_{101}, Pd_0.5_-TiO_2_{101} and Pd_0.5_-_cal_TiO_2_{101}.

Figure S16. Raman spectra of TiO_2_{101} and cal-Pd_0.05_/TiO_2_{101}.

Figure S17. (A) UV-DRS spectra and related (B) Tauc plot of Pd_0.025_-TiO_2_{101}, Pd_0.05_-TiO_2_{101}, Pd_0.075_-TiO_2_{101}, Pd_0.1_-TiO_2_{101}, Pd_0.3_-TiO_2_{101},Pd_0.5_/TiO_2_{101} and cal-Pd_0.5_/TiO_2_{101}.

Figure S18. Fluorescence spectra of TiO_2_{100}, TiO_2_{001}, TiO_2_{101} and Pd_0.05_/TiO_2_{101}

Figure S19. Time-decay fluorescence spectra of TiO_2_{101}, TiO_2_{001} and TiO_2_{100}.

Figure S20 Yields of photocatalytic CH_4_ conversion over TiO_2_{001}, TiO_2_{100} and TiO_2_{101}. Reaction condition: 10 mg of catalyst; CH_4_ : O_2_ = 100 : 1 at a flow rate of 10 mL·min^−1^; 70 W LED at 365 nm)

Figure S21. Photocurrent responses of TiO_2_{101}, Pd_0.05_/TiO_2_{101}, and cal-Pd_0.05_/TiO_2_{101}

Figure S22 XPS spectra of O 1s for cal-Pd_0.05_/TiO_2_{101}

The O 1s spectrum (Figure S22) was deconvoluted into two components: a dominant peak at 529.7 eV, attributed to lattice oxygen in stable, high-coordination environments, and a minor peak at 531.6 eV, attributed to surface oxygen species in lower-coordination environments, including bridging oxygen and hydroxyl groups. This deconvolution profile is essentially identical to that of bare TiO_2_{101}, demonstrating that Pd introduction at 0.05 wt% does not perturb the surface oxygen coordination environment.

Figure S23 XPS spectra of Ti 2p for cal-Pd_0.05_/TiO_2_{101}

The Ti 2p spectrum (Figure S23) shows dominant peaks at 458.8 eV (Ti^4+^ 2p_3/2_) and 464.2 eV (Ti^4+^ 2p_1/2_), fully consistent with Ti^4+^ in the anatase TiO_2_ lattice. A minor pair at 457.6 eV (Ti^3+^ 2p_3/2_) and 463.4 eV (Ti^3+^ 2p_1/2_) indicates the presence of a small concentration of Ti^3+^ species at the TiO_2_{101} surface. This observation is mechanistically significant, showing that Pd^2+^ species in the Pd 3d XPS spectrum are stabilized by direct coordination to lattice oxygen atoms of the TiO_2_{101} surface. A small fraction of Pd species at cluster–support interfaces induces localized Ti^3+^ formation via charge transfer, without compromising the overall lattice integrity of the TiO_2_ support.

Figure S24. In situ Pd 3d XPS spectra of cal-Pd_0.05_/TiO_2_{101} in dark and under light irradiation

Figure S25. Fluorescence spectra of cal-Pd_0.05_/TiO_2_{101} and Pd_0.9_/TiO_2_{101}

Figure S26. Photocurrent responses of cal-Pd_0.05_/TiO_2_{101} and Pd_0.9_/TiO_2_{101}

Figure S27. CH_4_ temperature-programmed desorption over TiO_2_{001}, TiO_2_{100} and TiO_2_{101}

Figure S28. Raman spectra of TiO_2_{101} and TiO_2_{001}

Figure S29. O1s XPS spectra of TiO_2_{101} and TiO_2_{001}

Figure S30. Raman images of cal-Pd_0.05_/TiO_2_{101} after reaction for 1-5 h.

Figure S31. Raman spectra of Cal-Pd_0.05_/TiO_2_{101} after reaction for 1-5 h.

Figure S32. FT-IR spectra of fresh cal-Pd_0.05_/TiO_2_{101} and after the CH_4_ oxidation for 5 h.

Figure S33. BET analysis of (A) fresh cal-Pd_0.05_/TiO_2_{101} catalyst and (B) cal-Pd_0.05_/TiO_2_{101} catalyst after CH_4_ conversion.

Figure 34. XRD patterns of fresh cal-Pd_0.05_/TiO_2_{101} catalyst and cal-Pd_0.05_/TiO_2_{101} catalyst after CH_4_ conversion.

Figure S35. (A) UV-DRS spectra and related (B) Tauc plot of fresh cal-Pd_0.05_/TiO_2_{101} catalyst and cal-Pd_0.05_/TiO_2_{101} catalyst after CH_4_ conversion.

Figure S36. In-situ DRIFTS of CH_4_ oxidation over cal-Pd_0.05_/TiO_2_{101}

**Supplementary table**

Table S1. Pd content of catalysts.

| sample | Content of Pd (%) |
| --- | --- |
| Pd_0.025_/TiO_2_{101} | 0.026 |
| Pd_0.05_/TiO_2_{101} | 0.048 |
| Pd_0.075_/TiO_2_{101} | 0.076 |
| Pd_0.1_/TiO_2_{101} | 0.110 |
| Pd_0.3_/TiO_2_{101} | 0.305 |
| Pd_0.5_/TiO_2_{101} | 0.512 |

Table S2. Representative works on photocatalytic CH_4_ conversion to C_2_H_6_^[4-6, 18,40-52]^.

| catalyst | Reaction condition | C_2_H_6_ production (μmol/h) | C_2_H_6_ selectivity | Stability | Ref. |
| --- | --- | --- | --- | --- | --- |
|  | | | | | |
| cal-Pd_0.05_/TiO_2_{101} | 10 mg catalyst, pure CH_4_ , 0.023 W·cm^-2^ | 6.1 (610 μmol·g^-1^·h^-1^) | nearly 100% | Drops to 90 % over 30 h | This work |
| Pd_1_/TiO_2_ | 3 mg catalyst, pure CH_4_, 0.6 W·cm^-2^ | 2.75 (917 μmol·g^-1^·h^-1^) | 94.3 % | Drops to 20% over 6 h | 11 |
|  | 3 mg catalyst,  10 vol % CH_4_/Ar, 0.6 W·cm^-2^ | 0.86 (287 μmol·g^-1^·h^-1^) | No report  (NR) |  |  |
|  | 50 mg catalyst, pure CH_4_, 0.6 W·cm^-2^ | 40 (800 μmol·g^-1^·h^-1^) | 85 % |  |  |
| ^a^ Pt/Ga_2_O_3_ | 200 mg catalyst, pure CH_4_, | 0.11 (0.55 μmol·g^-1^·h^-1^) | 97 % | Drops to 50% over 24 h | 25 |
| Zn-ZSM-5 | 1000 mg catalyst, pure CH_4_, 0.1 W·cm^-2^ | 3 (3 μmol·g^-1^·h^-1^) | 100 % | NR | 26 |
| Pd-Au-ZnO | 2 mg catalyst, pure CH_4_, 0.6 W·cm^-2^ | 0.04 (20 μmol·g^-1^·h^-1^) | 53 % | Drops to 60% over 16 h | 27 |
| Au/m-ZnO | 1 mg catalyst, 1.25 vol % CH_4_/Ar, 0.6 W·cm^-2^ | 0.001 (1 μmol·g^-1^·h^-1^) | 100 % | Drops to 10% over 7 h | 28 |
| Pt/HGTS | 200 mg catalyst, pure CH_4_, 0.18 W·cm^-2^ | 0.32 (1.6 μmol·g^-1^·h^-1^) | 90.1 % | Drops to 52% over 16 h | 29 |
| Nb-TS | 100 mg catalyst, pure CH_4_, | 0.17 (1.7 μmol·g^-1^·h^-1^) | 96.2 % | Drops to 76% over 16 h | 30 |
| Ce_(x)_/Al_2_O_3_ | 200 mg catalyst, pure CH_4_, 0.07 W·cm^-2^ | 0.19 (0.95 μmol·g^-1^·h^-1^) | 77.6 % | NR | 31 |
| GaN:ZnO | 50 mg catalyst, pure CH_4_, 0.6 W·cm^-2^ | 1.45 (29 μmol·g^-1^·h^-1^) | > 89 % | NR | 32 |
|  | 2 mg catalyst, pure CH_4_, 0.6 W·cm^-2^ | 0.33 (165 μmol·g^-1^·h^-1^) | NR |  |  |
| Au/TiO_2_ | 5 mg catalyst,  10 vol % CH_4_/Ar, 0.1 W·cm^-2^ | 0.41 (82 μmol·g^-1^·h^-1^) | 95 % | Drops to 80% over 16 h | 33 |

^a^ The reported C_2_H_6_ rate was calculated from Pt per unit mass.

The thermodynamic constraint on non-oxidative methane conversion (NOMC) was evaluated based on the standard Gibbs free energy change (ΔG) of the following representative C_2_-forming reaction:

2CH_4_(g)→C_2_H_6_(g)+H_2_(g)

The standard Gibbs free energy change was calculated according to:

ΔG=ΔH−TΔS

Where the standard enthalpy change (ΔH) and standard entropy change (ΔS) were determined from:

ΔH=Σ ΔH_f_(products)-Σ ΔH_f_(reactants)

ΔS=Σ S(products)-Σ S(reactants)

Table S3. Standard Thermodynamic Data for Species Involved in the Non-Oxidative Methane Conversion (NOMC) Reaction at T = 298 K [Chase, M. (1998), NIST-JANAF Thermochemical Tables, 4th Edition, American Institute of Physics.]

| species | ΔH_f_ (kJ/mol) | S (J/mol·K) |
| --- | --- | --- |
| CH_4_ (g) | -74.8 | 186.3 |
| C_2_H_6_ (g) | -83.8 | 229.6 |
| H_2_ (g) | 0 | 130.7 |

ΔH=+65.8 kJ/mol

ΔS=−12.3 J/mol·K

ΔG≈+68.6 kJ/mol

**Reference**

[11]	W. Zhang, C. Fu, J. Low, D. Duan, J. Ma, W. Jiang, Y. Chen, H. Liu, Z. Qi, R. Long, Y. Yao, X. Li, H. Zhang, Z. Liu, J. Yang, Z. Zou, Y. Xiong (2022) High-performance photocatalytic nonoxidative conversion of methane to ethane and hydrogen by heteroatoms-engineered TiO_2_. Nature communications, 13, 2806.

[25]	J. Ma, X. Tan, Q. Zhang, Y. Wang, J. Zhang, L. Wang (2021) Exploring the Size Effect of Pt Nanoparticles on the Photocatalytic Nonoxidative Coupling of Methane. ACS Catalysis, 11, 3352-3360.

[26]	L. Li, G.-D. Li, C. Yan, X.-Y. Mu, X.-L. Pan, X.-X. Zou, K.-X. Wang, J.-S. Chen (2011) Efficient Sunlight-Driven Dehydrogenative Coupling of Methane to Ethane over a Zn^+^-Modified Zeolite. Angewandte Chemie International Edition, 50, 8299-8303.

[27]	W. Jiang, J. Low, K. Mao, D. Duan, S. Chen, W. Liu, C.-W. Pao, J. Ma, S. Sang, C. Shu, X. Zhan, Z. Qi, H. Zhang, Z. Liu, X. Wu, R. Long, L. Song, Y. Xiong (2021) Pd-Modified ZnO–Au Enabling Alkoxy Intermediates Formation and Dehydrogenation for Photocatalytic Conversion of Methane to Ethylene. Journal of the American Chemical Society, 143, 269-278.

[28]	L. Meng, Z. Chen, Z. Ma, S. He, Y. Hou, H.-H. Li, R. Yuan, X.-H. Huang, X. Wang, X. Wang, J. Long (2018) Gold plasmon-induced photocatalytic dehydrogenative coupling of methane to ethane on polar oxide surfaces. Energy & Environmental Science, 11, 294-298.

[29]	S. Wu, X. Tan, J. Lei, H. Chen, L. Wang, J. Zhang (2019) Ga-Doped and Pt-Loaded Porous TiO_2_–SiO_2_ for Photocatalytic Nonoxidative Coupling of Methane. Journal of the American Chemical Society, 141, 6592-6600.

[30]	Z. Chen, S. Wu, J. Ma, S. Mine, T. Toyao, M. Matsuoka, L. Wang, J. Zhang (2021) Non-oxidative Coupling of Methane: N-type Doping of Niobium Single Atoms in TiO_2_–SiO_2_ Induces Electron Localization. Angewandte Chemie International Edition, 60, 11901-11909.

[31]	L. Yuliati, T. Hamajima, T. Hattori, H. Yoshida (2008) Nonoxidative Coupling of Methane over Supported Ceria Photocatalysts. The Journal of Physical Chemistry C, 112, 7223-7232.

[32]	G. Wang, X. Mu, J. Li, Q. Zhan, Y. Qian, X. Mu, L. Li (2021) Light-Induced Nonoxidative Coupling of Methane Using Stable Solid Solutions. Angewandte Chemie International Edition, 60, 20760-20764.

[33]	J. Lang, Y. Ma, X. Wu, Y. Jiang, Y. H. Hu (2020) Highly efficient light-driven methane coupling under ambient conditions based on an integrated design of a photocatalytic system. Green Chemistry, 22, 4669-4675.
